# Dry Eye Parameters and Lid Geometry in Adults Born Extremely, Very, and Moderately Preterm with and without ROP: Results from the Gutenberg Prematurity Eye Study

**DOI:** 10.3390/jcm11102702

**Published:** 2022-05-11

**Authors:** Achim Fieß, Clara Hufschmidt-Merizian, Sandra Gißler, Ulrike Hampel, Eva Mildenberger, Michael S. Urschitz, Fred Zepp, Bernhard Stoffelns, Norbert Pfeiffer, Alexander K. Schuster

**Affiliations:** 1 Department of Ophthalmology, University Medical Center of the Johannes Gutenberg University Mainz, 55131 Mainz, Germany; clara.hufschmidt-merizian@gast.unimedizin-mainz.de (C.H.-M.); sandra.gissler@unimedizin-mainz.de (S.G.); bernhard.stoffelns@unimedizin-mainz.de (B.S.); norbert.pfeiffer@unimedizin-mainz.de (N.P.); alexander.schuster@unimedizin-mainz.de (A.K.S.); 2 Department of Ophthalmology, University Hospital Leipzig, 04103 Leipzig, Germany; uli.hampel@medizin.uni-leipzig.de; 3 Division of Neonatology, Department of Pediatrics, University Medical Center of the Johannes Gutenberg University Mainz, 55131 Mainz, Germany; eva.mildenberger@unimedizin-mainz.de (E.M.); zepp@uni-mainz.de (F.Z.); 4 Division of Pediatric Epidemiology, Institute for Medical Biostatistics, Epidemiology and Informatics, University Medical Center of the Johannes Gutenberg University Mainz, 55131 Mainz, Germany; urschitz@uni-mainz.de

**Keywords:** Schirmer test, dry eye, sicca, prematurity, retinopathy of prematurity, epidemiology

## Abstract

Background/Aims: This study aimed to analyze the effects of perinatal history on tear film properties and lid geometry in adults born preterm. Methods: The Gutenberg Prematurity Eye Study (GPES) is a German prospective examination of adults born preterm and term aged 18 to 52 years with Keratograph^®^ 5M and Schirmer test I. Main outcome measures were first non-invasive tear film break-up time (F-NITBUT), bulbar redness (BR), Schirmer test, and nasal palpebral angle measurement. The associations with gestational age (GA), birth weight (BW), and BW percentile, retinopathy of prematurity (ROP), ROP treatment, and other perinatal factors were evaluated using regression analyses. Results: 489 eyes of 255 preterm and 277 eyes of 139 full-term individuals (aged 28.6 +/− 8.8 years, 220 females) were included. Of these, 33 participants (56 eyes) had a history of spontaneously regressed ROP and 9 participants (16 eyes) had a history of ROP treatment. After adjustment for age and sex, lower F-NITBUT (<20 s) was associated with ROP treatment (OR = 4.42; *p* = 0.025). Lower GA correlated with increased bulbar redness (B = −0.02; *p* = 0.011) and increased length of wetting in the Schirmer test (B = −0.69; *p* = 0.003). Furthermore, low GA was associated with narrowing of the nasal palpebral angle (B = 0.22; *p* = 0.011) adjusted for age and sex, but not when considering ROP in the multivariable model. Conclusion: Our analyses indicate that perinatal history affects ocular surface properties, tear production and lid geometry in adults born term and preterm. This might indicate that affected persons have a predisposition to diseases of the corneal surface such as the dry eye disease.

## 1. Introduction

Preterm delivery leads to an abrupt change in the surrounding fetal environment and may result in ocular changes, especially in those with immediate exposure to the environment such as the ocular surface. Indeed, children born preterm have altered ocular morphology, including a steeper corneal geometry [1], a smaller anterior chamber depth [2], thicker lens [2], shorter axial length [1,2], and altered posterior pole [3,4,5]. Similar results were reported in adults indicating that low birth weight (<2500 g) as a surrogate marker for prematurity is associated with steeper corneas and shorter axial lengths [6]. These results show that the effects of prematurity on ocular shape persist throughout life. Furthermore, extreme preterm newborns are at increased risk for the postnatal development of retinopathy of prematurity (ROP) which is a vasoproliferative disease of the retina caused by high oxygen levels and the major cause for reduced visual acuity in childhood [7,8]. Several reports observed that ROP is an additional parameter affecting corneal shape in children [1]. Additionally, the corneal surface is less regular as indicated by increased corneal aberrations measured in preterm children [9] and adults [10]. This is of particular importance because every year about 15 million newborns are born preterm globally and the prevalence of ROP has still been increasing for several decades worldwide [7,11].

There are different hypotheses and models explaining the effects of an altered corneal geometry. For example, Fielder et al. hypothesized that the lower extrauterine temperature leads to a decreased flattening of the cornea after preterm birth [12], whereas other researchers supposed that a shorter time in the intrauterine milieu and different periods of opened eyes [13] may result in ultrastructural corneal remodeling processes including collagen fiber layers. Impaired prenatal growth also leads to altered organ morphology and functioning [14], as well as increased inflammation in a hyper-responsive innate immune system [15]. Until now, it is unclear whether this pro-inflammatory tendency after premature birth and different environmental exposure may also influence the ocular surface in which subacute inflammation is a risk factor for homeostasis and may lead to dry eye disease. Previous reports demonstrated that dry eye disease has many features in common with autoimmune disease [16], which may lead to an increased risk of dry eye disease in preterm individuals.

Dry eye disease is a multifactorial disease of the tears and ocular surface leading to discomfort, an unstable tear film, and visual disturbance [17], with up to every third person worldwide suffering from dry eye disease [18]. In recent decades, many studies investigated triggers of dry eye disease such as environmental factors, endogenous stress, antigens, infections, and genetic factors [16,19]. Another risk factor may be the perinatal history, potentially leading to life-long alterations of the ocular surface. As prematurity is linked to altered ocular geometry, there is the possibility that perinatal history may also affect eyelid formation and ocular surface characteristics. Hence, this investigation assessed the status of the ocular surface by measuring the tear film break-up time, bulbar redness, length of wetting the Schirmer test I, and nasal palpebral angle of subjects born preterm at different gestational ages (GA) with and without ROP compared to full-term controls now aged between 18 to 52 years.

**Precis:**  This study investigated the long-term effects of prematurity on dry eye disease parameters and lid geometry in adulthood. The results indicate that the more preterm individuals are born, the more frequently alterations of ocular surface occur.

**Key messages of the article:**
  − What is already known on this topic?
    Prematurity is associated with altered ocular morphology and functioning in childhood and adulthood, so we investigated whether perinatal factors have long-term effects on the ocular surface and lid configuration in adulthood.
  − What does this study add?
    ROP treatment is linked to reduced tear film break-up time later in life. Furthermore, low gestational age is associated with increased bulbar redness, longer Schirmer strip measurement, and a narrower nasal lid angle.
  − How might this study affect research or practice?
    Perinatal history affects the ocular surface, tear production, and lid geometry in adults born term and preterm, which might predispose affected persons to diseases of the corneal surface and dry eye disease in later life.

## 2. Materials and Methods

### 2.1. Study Population

The Gutenberg Prematurity Eye Study (GPES) is a single-center cohort study at the University Medical Center of the Johannes Gutenberg University Mainz in Germany (UMCM) that recruits individuals that (i) have been born preterm or at term between 1969 and 2002 and (ii) were between 18 and 52 years of age at study enrolment. According to these design elements, the study is a retrospective cohort study with a prospective acquisition of follow-up data. For the GPES, every preterm newborn with a GA ≤ 32 weeks and every second randomly chosen preterm newborn with GA 33–36 weeks was contacted and invited to participate. From each month from 1969 to 2002, 6 (3 males and 3 females) randomly selected full-term subjects with a birth weight between the 10th and 90th percentile were also invited to serve as controls as reported earlier [20,21,22,23,24].

The study examinations were performed between 2019 and 2021. The flow chart for eligibility and recruitment efficacy proportion is shown in Figure S1. Every participant underwent a detailed ophthalmological examination including measurement with a Keratograph^®^ 5M (Oculus, Wetzlar, Germany) and a medical history interview. Furthermore, their medical records documenting the perinatal and postnatal history were assessed.

Written informed consent was obtained from all participants before their entry into the study and the GPES complies with Good Clinical Practice (GCP), Good Epidemiological Practice (GEP), and the ethical principles of the Declaration of Helsinki. The study protocol and documents were approved by the local ethics committee of the Medical Chamber of Rhineland-Palatinate, Germany (reference no. 2019-14161; original vote: 29 May 2019, latest update: 2 April 2020).

### 2.2. Assessment of Pre-, Peri- and Postnatal Medical History

The medical histories were assessed from medical records stored at the UMCM. Data were collected regarding GA (weeks), birth weight (kg), presence of ROP, stage of ROP, ROP treatment, placental insufficiency, preeclampsia, maternal smoking during pregnancy and breastfeeding. Birth weight percentiles were also calculated according to Voigt et al. [25].

### 2.3. Categorization

For descriptive analysis, participants were allocated to group 1: full-term participants (GA ≥ 37 weeks), group 2: preterm participants with GA between 33–36 weeks without ROP, group 3: preterm participants with GA between 29–32 weeks without ROP, group 4: preterm participants with GA ≤ 28 weeks without ROP, group 5: preterm participants with GA ≤ 32 weeks with postnatal ROP without ROP treatment and group 6: preterm participants with GA ≤ 32 weeks with postnatal ROP and ROP treatment as reported earlier [21,22]. In the case that only one eye of a participant had ROP, the other non-ROP eye was excluded from the analysis.

### 2.4. Ophthalmological Examination

Each participant was examined with a Keratograph^®^ 5M (Oculus, Wetzlar, Germany) to measure the non-invasive tear film break-up time (first: F-NITBUT) and grade of break-up time. Furthermore, the software of the Keratograph^®^ 5M measures the nasal and temporal conjunctival areas, detects conjunctival blood vessels and calculates redness grade. Values for the mean global bulbar redness consist of values from temporal and nasal bulbar redness. The limbal nasal and limbal temporal redness values are subcategories of the nasal and temporal bulbar redness, respectively. Lid geometry was determined by analyzing photographs of the anterior segment and measuring the palpebral fissure and the nasal palpebral angle. Each parameter was controlled for outliers.

Tear production was measured by the Schirmer test. In every participant, the distance of the tears to travel along the length of a paper test strip was measured [1] without anesthesia of the ocular surface (Schirmer test I). Furthermore, the study participants completed the ocular surface and disease questionnaire, and the Ocular Surface Disease Index (OSDI) was calculated [26].

### 2.5. Covariates

Covariates were factors that may have affected the main outcome measures such as sex (female), age (years), GA (weeks), birth weight (gram), birth weight percentile, ROP (yes), ROP treatment (yes), placental insufficiency (yes), preeclampsia (yes), breastfeeding (yes), and maternal smoking during pregnancy (yes).

### 2.6. Inclusion/Exclusion Criteria

Only participants with successful measurement of the tear film break-up time were included. Each measurement was checked for correctness and centration and excluded if considered invalid. Participants with a history of corneal or cataract surgery were excluded as this may have contributed to an altered ocular surface.

### 2.7. Statistical Analysis

The main outcome measures were non-invasive tear film break-up time, bulbar redness, length of wetting in the Schirmer test I, and nasal palpebral angle. Descriptive statistics were computed for these measures stratified by clinical group. Absolute and relative frequencies were calculated for dichotomous parameters; the mean and standard deviation were calculated for approximately normally distributed variables, otherwise median and interquartile range. Categorical data were compared with the chi-square test, with not-normally distributed continuous parameters compared with the Mann–Whitney U-test. Linear regression models in the case of normal distribution with general estimating equations (GEE) were used to assess associations and account for correlations between corresponding eyes. For non-normally distributed parameters, quantile regression was performed including only right eyes (Schirmer I test). Binary logistic regression analyses were conducted for reduced F-NITBUT (<20 s). First, univariate analyses of the main outcome measures and sex (female), age (years), GA (weeks), birth weight (kg), birth weight percentile, ROP (yes), ROP treatment (yes), placental insufficiency (yes), preeclampsia (yes), breastfeeding (yes) and maternal smoking during pregnancy (yes) were computed. Then, only parameters associated in the univariate analyses were included in model #1 with additional adjustment for sex and age. In a further model, the potential effect of ROP occurrence (yes) and/or ROP treatment (yes) were analyzed. Birth weight was excluded in the multivariable models to avoid collinearity which was strong between GA and birth weight. This was an explorative study, so there was no adjustment for multiple testing. Calculations were performed using commercial software (IBM SPSS 20.0; SPSS, Inc., Chicago, IL, USA; R version 4.0.0 (24 April 2020), R Core Team (2020), R: A language and environment for statistical computing and R Foundation for Statistical Computing, Vienna, Austria. URL https://www.R-project.org/; package “quantreg” (accessed 10 January 2022).

## 3. Results

### 3.1. Participant Characteristics

The present analysis included 489 eyes of 255 preterm and 277 eyes of 139 full-term individuals (age 28.6 +/− 8.8 years, 220 females). Overall, there were 277 eyes of 139 participants with GA ≥ 37 weeks (group 1), 256 eyes of 129 participants with a GA between 33–36 weeks without ROP (group 2), 133 eyes of 70 participants with a GA between 29–32 weeks without ROP (group 3), 28 eyes of 14 participants with a GA ≤ 28 weeks without ROP (group 4), 56 eyes of 33 participants with a GA between 24–32 weeks with ROP without treatment (group 5), and 16 eyes of 9 participants with a GA between 24–32 and with postnatal treatment for ROP (group 6). Of the ROP-treated group, five (nine eyes) participants underwent laser coagulation, while four (seven eyes) participants had cryocoagulation. The recruitment efficacy proportion for each group is presented in Supplementary Figure S1. Overall, 7 participants were excluded because of previous corneal refractive surgery, cataract surgery, or ocular trauma, and further 40 participants were excluded because measurement with a keratograph was invalid or not possible. In total, eight eyes without ROP were excluded in which the fellow eye had postnatal ROP. The participants’ characteristics are presented in Table 1.

### 3.2. Descriptive Anterior Surface Parameters

**Figure 1:**
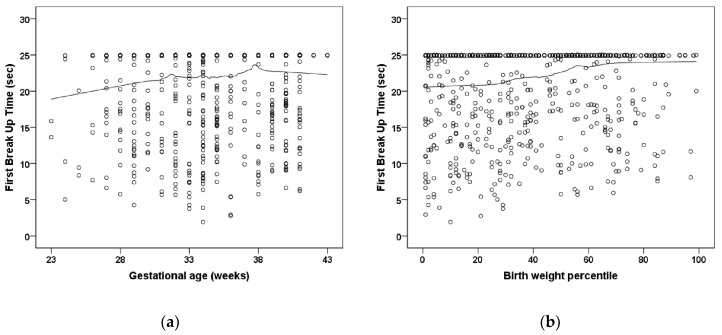
Relationship of (**a**) gestational age and (**b**) birth weight percentile to the first break-up time (sec.) in the Gutenberg Prematurity Eye Study. Figure legend 1: There is no significant association between low gestational age and low birth weight percentile with first break-up time. The line presents the Loess (Locally Weighted Scatterplot Smoothing) curve.

**Figure 2:**
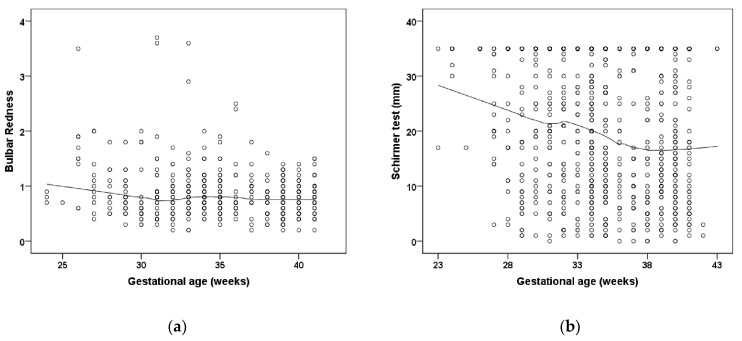
The relationship of gestational age to (**a**) bulbar redness, and (**b**) wetting in the Schirmer test in the Gutenberg Prematurity Eye Study. Figure legend 2: Individuals born preterm with low gestational age show an increased bulbar redness and a higher Schirmer test result. The line presents the Loess (Locally Weighted Scatterplot Smoothing) curve.

**Figure 3:**
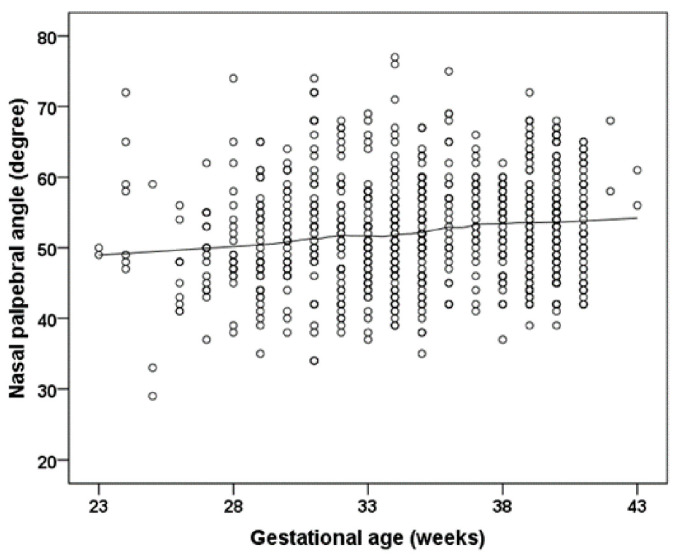
The relationship of gestational age to the nasal palpebral angle. Figure legend 3: Participants with lower gestational age reveal a smaller nasal palpebral angle. The line presents the Loess (Locally Weighted Scatterplot Smoothing) curve.

Participants treated for ROP showed a lower F-NITBUT (*p* = 0.032; Table 2) (Figure 1). Bulbar redness was increased in the preterm group with a GA 33–36 weeks (*p* = 0.034; group 2) and the group with untreated ROP participants (*p* = 0.024; group 5) and in the ROP treated participants (*p* = 0.048; group 6) compared to the full-term control group (group 1) (Table 2). Furthermore, the measurement in the Schirmer test was significantly increased in the preterm groups with a GA 33–36 weeks (*p* = 0.001; group 2), GA 29–32 weeks without ROP (*p* = 0.001; group 3), and GA ≤ 28 weeks without ROP (*p* < 0.001; group 4), and descriptively increased in participants with GA ≤ 32 weeks with ROP without treatment (*p* = 0.092; group 5) and with ROP treatment (*p* = 0.054; group 6) compared to the full-term control group (group 1) (Table 2; Figure 2). Parameters for palpebral fissure and angle are displayed in Table 3 and Figure 3.

### 3.3. Uni- and Multivariable Analyses

In the association analyses, first univariable analyses were performed. Associated parameters were then included in model #1 without the inclusion of ROP occurrence and ROP treatment. In a second multivariable model, ROP occurrence and treatment were additionally included if they were associated in the univariable model. Models #1 and #2 were additionally adjusted for sex and age. First tear film break-up time (<20 s) correlated in univariable analyses with ROP occurrence (*p* = 0.024) and ROP treatment (*p* = 0.009). After adjustment for sex and age and the additional inclusion of ROP occurrence and treatment, ROP treatment (OR = 4.42 [95% CI: 1.20; 16.28]; *p* = 0.025) but not ROP occurrence was associated with F-NITBUT < 20 s. In the univariable analyses, Bulbar redness was associated with GA (*p* = 0.004), birth weight (*p* = 0.003), and ROP occurrence (*p* = 0.05). After adjustment for sex and age in model #1, GA (*p* < 0.001) was still associated with bulbar redness. In model #2, a low GA revealed an association (B = −0.015 [95% CI: −0.027; −0.003]; *p* = 0.011) with bulbar redness but not ROP occurrence.

The measurement in the Schirmer test was associated with GA (*p* < 0.001), birth weight (<0.001), and placental insufficiency (*p* = 0.029) in univariable analyses. In multivariable model #1, the Schirmer test results were still associated with gestational age (B = −0.692 [95% CI: −1.158; −0.226] mm; *p* = 0.003) and placental insufficiency (B = 8.596 [95% CI: 4.643; 12.55] mm; *p* < 0.001).

Nasal palpebral angle correlated with GA (*p* = 0.002), birth weight (*p* < 0.002), ROP occurrence (*p* = 0.002), and preeclampsia (*p* = 0.040). After adjustment for sex and age, GA (*p* = 0.011) and preeclampsia (*p* = 0.034) showed both associations. In model #2, GA and ROP treatment showed no association, while preeclampsia was associated (B = 2.492 [95% CI: 0.320; 4.664] mm; *p* = 0.025) (Table 4).

## 4. Discussion

The present study provides new data describing the long-term effects of prematurity, ROP, and perinatal factors on ocular surface health in adults born preterm. Our study shows that prematurity correlated with an increase in bulbar redness, increased length of wetting in the Schirmer test, and a narrower nasal palpebral angle. Furthermore, ROP treatment was associated with a tear film break-up time lower than 20 s. Overall, this data indicates that adults born preterm may have altered ocular surface homeostasis.

This study provided new insights regarding the long-term effects of perinatal history on ocular surface characteristics. It is well known that preterm birth and the postnatal occurrence of ROP are linked to an altered organ morphology in childhood, adolescence, and adulthood. The Wiesbaden Prematurity Study observed that prematurity was linked with a steeper corneal curvature, while postnatal ROP occurrence and treatment were linked to a shorter anterior chamber depth [1]. In congruence, a population-based report analyzing adolescents showed an association between lower birth weight as a proxy for prematurity and steeper corneal curvature [27]. Recent reports indicate that these alterations persist throughout life, as results from the Gutenberg Health Study showed that low birth weight (<2500 g) was associated with a steeper corneal radius, smaller corneal diameter [6], and altered posterior pole [28,29,30,31]. Furthermore, other studies reported a less regular corneal surface in subjects born preterm with low birth weight, as indicated by increased corneal aberrations in childhood and adulthood [9,10]. The present results indicate that prematurity affects not only ocular geometry but also other ocular surface properties such as tear film quantity and stability.

While Raffa and colleagues [32] observed no association between the eyelid aperture in individuals born preterm and term aged 4 to 15 years, the present study found a narrower nasal palpebral angle in individuals born preterm. One may speculate that this is an unknown risk factor potentially influencing ocular refractive and geometric development in individuals born preterm, as previous reports observed that palpebral angles and lid aperture are risk factors for spherical refractive error development [33,34].

Individuals affected by dry eye diseases suffer from ocular discomfort, burning, instable tear film, and visual disturbance that can lead to inflammatory damage of the ocular surface [17]. They may also have reduced quality of life [35] and lower vision-related quality of life [36] with a prevalence up to one in three in the general population [37] with a high economic impact [38]. There are different recognized risk factors, such as environmental factors, endogenous stress, antigens, infections, genetic factors, and autoimmune disorders [16], which only partially explain the occurrence of dry eye disease, so other risk factors may be involved. Our data are the first to suggest that prematurity and associated factors may predispose to dry eye disease later in life. Dry eye disease can be classified as decreased tear secretion or increased tear evaporation. Since the preterm participants revealed significantly increased Schirmer test measures, one may speculate that preterm birth leads to tear overproduction accompanied by a less stable tear film. However, the pathophysiological mechanisms remain unclear, and it remains to be determined whether the increased tear evaporation is caused by dysfunction of the Meibomian glands or by reduced mucin from the goblet cells. Other pathophysiological mechanisms causing long-term effects on the corneal surface might be the lower extrauterine temperature after preterm birth compared to the intrauterine environment [12] and a shorter time in the intrauterine milieu, or different periods of opened eyes in the interval until reaching full GA [13]. There is evidence that dry eye disease is caused, among other reasons, by subacute inflammation and has many features in common with autoimmune disease [16]. Prematurity is linked to a hyper-responsive innate immune system [15]; thus, one may speculate that this pro-inflammatory tendency also influences the ocular surface. In addition, participants being treated for postnatal ROP revealed a shorter tear film break-up time also potentially indicating that absorbed energy during treatment leads to life-long alterations of corneal hemostasis. It is well known that prematurity is a risk factor for psychosomatic diseases, such as depression, which is also a risk factor for dry eye disease [39]. However, the clinical significance of our findings remains unclear because we observed no difference in the OSDI score between the different study groups. It is possible that the observed ocular changes are of subclinical importance and might predispose to dry eye diseases in later life.

## 5. Strengths and Limitations

One limitation of the present study is the single-center hospital-based study design. Furthermore, other lid parameters were not investigated, such as frequency of blinking in different environments, horizontal lid aperture, and temporal lid aperture. Invalid or missing keratograph examinations may further limit the present data. Moreover, the number of individuals with a history of ROP and ROP treatment was low, which must be considered during the data interpretation. A further limitation is the missing data about tissue status and function of the meibomian gland in the present study which should be examined in future studies.

The strengths are that this study was one of the largest cohorts of adults born preterm at different GA with and without ROP. Detailed assessment of perinatal history from medical records enabled a comprehensive data analysis of the perinatal effects on the ocular surface, with all measurements and validation steps blinded with respect to GA and other perinatal factors.

## 6. Conclusions

In conclusion, our analyses indicate that perinatal history affects the ocular surface later in life. Preterm birth was associated with increased bulbar redness, increased length of wetting in the Schirmer test, and a narrower palpebral angle, while ROP treatment was associated with a reduced tear film break-up time of less than 20 s, suggesting that affected persons may be predisposed to diseases of the corneal surface in later life, such as dry eye disease.

## Figures and Tables

**Table 1:** Characteristics of the sample of the Gutenberg Prematurity Eye Study stratified by study groups.

	Group 1	Group 2	Group 3	Group 4	Group 5	Group 6
Gestational Age [Weeks]	GA ≥ 37	GA 33–36	GA 29–32	GA ≤ 28	GA ≤ 32	GA ≤ 32
		no ROP	no ROP	no ROP	ROP	ROP with Treatment
Participants/eyes (n)	139/277	129/256	70/133	14/28	33/56	9/16
Sex (Women) (%)	81 (58.3%)	77 (59.7%)	37 (52.9%)	8 (57.1%)	15 (45.5%)	2 (22.2%)
Age (y)	29.9 ± 9.2	29.3 ± 9.2	27.8 ± 8.1	23.9 ± 8.2	24.5 ± 5.3	27.9 ± 6.3
Birth weight (g)	3420 ± 393	2064 ± 473	1587 ± 347	930 ± 218	1120 ± 392	862 ± 304
Birth weight < 1500 g (yes)	0 (0%)	13 (10.1%)	28 (40%)	14 (100%)	28 (84.8%)	9 (100%)
Birth weight < 1000 g (yes)	0 (0%)	0 (0%)	4 (5.7%)	7 (50%)	12 (36.4%)	6 (66.7%)
Birth weight percentile	48.7 ± 21.4	25.2 ± 24.5	45.2 ± 23.8	41.1 ± 24.6	37.7 ± 29.4	24.2 ± 27.1
Gestational age (wks)	39.3 ± 1.3	34.3 ± 1.0	30.7 ± 1.1	26.8 ± 1.6	28.4 ± 1.9	27.4 ± 2.7
(min–max)	(37–43)	(33–36)	(29–32)	(23–28)	(24–32)	(24–32)
ROP stage (1/2/3/4/5)	0/0/0/0/0	0/0/0/0/0	0/0/0/0/0	0/0/0/0/0	25/25/5/0/1	0/2/14/0/0
Preeclampsia (yes)	11 (7.9%)	23 (17.8%)	8 (11.4%)	2 (14.3%)	7 (21.2%)	2 (22.2%)
Placental insufficiency (yes)	2 (1.4%)	16 (12.4%)	1 (1.4%)	1 (7.1%)	2 (6.1%)	0 (0%)
HELLP-syndrome	0 (0%)	6 (4.7%)	0 (0%)	0 (0%)	4 (12.1%)	0 (0%)
Maternal smoking (yes)	7 (5%)	7 (5.4%)	5 (7.1%)	1 (7.1%)	3 (9.1%)	1 (11.1%)
Gestational diabetes (yes)	1 (0.7%)	6 (4.7%)	1 (1.4%)	0 (0%)	1 (3%)	0 (0%)
Breastfeeding (yes)	78 (56.1%)	69 (53.5%)	37 (52.9%)	7 (50%)	16 (48.5%)	5 (55.6%)
**Ocular parameters**						
Visual acuity (logMAR) OD	0.0 (0.0; 0.0)	0.0 (0.0; 0.0)	0.0 (0.0; 0.0)	0.0 (0.0; 0.0)	0.0 (0.0; 0.1)	0.0 (0.0; 0.3)
Visual acuity (logMAR) OS	0.0 (0.0; 0.0)	0.0 (0.0; 0.0)	0.0 (0.0; 0.0)	0.0 (0.0; 0.0)	0.0 (0.0; 0.0)	0.1 (0.0; 0.7)
Spherical equivalent (diopter) OD	−0.98 ± 2.2	−1.10 ± 2.20	−0.59 ± 2.19	−0.9 ± 2.53	−1.52 ± 3.39	−5.12 ± 7.51
Spherical equivalent (diopter) OS	−0.97 ± 2.09	−1.16 ± 2.18	−0.63 ± 2.19	−0.45 ± 2.19	−1.75 ± 3.41	−4.26 ± 10.98
Intraocular pressure (mmHg) OD	15.3 ± 2.8	14.7 ± 2.9	14.6 ± 3.3	16.1 ± 3.0	15.8 ± 3.5	16.0 ± 4.1
Intraocular pressure (mmHg) OS	15.2 ± 2.8	14.5 ± 3.0	14.5 ± 3.1	14.7 ± 3.0	16.3 ± 3.7	17.0 ± 4.4

g—gram; mm–millimeter; ROP—retinopathy of prematurity; dpt—diopter; OD—right eye; OS—left eye.

**Table 2:** Anterior segment parameters of the study groups.

	Group 1	Group 2	Group 3	Group 4	Group 5	Group 6
Gestational Age [Weeks]	GA ≥ 37	GA 33–36	GA 29–32	GA ≤ 28	GA ≤ 32	GA ≤ 32
		no ROP	no ROP	no ROP	ROP	ROP with Treatment
Participants/eyes (n)	139/277	129/256	70/133	14/28	33/56	9/16
** Break-up time **						
First BUT OD	20.7 ± 5.8	20.7 ± 6.2	20.2 ± 6.8	21.4 ± 5.4	19.6 ± 6.2	14.9 ± 7.8 #
First BUT OS	21.6 ± 5.2	19.6 ± 6.8 #	20.9 ± 5.9	20.3 ± 5.1	20.3 ± 5.1	12.2 ± 7.6 #
BUT ≤ 20 s OD + OS	89 (33.0%)	90 (36.0%)	41 (32.3%)	10 (37.0%)	21 (42.0%)	10 (71.4%) #
BUT ≤ 10 s OD + OS	21 (7.8%)	29 (11.6%)	14 (11.0%)	1 (3.7%)	3 (6.0%)	6 (42.9%) #
BUT ≤ 5 s OD + OS	0 (0%)	6 (2.4%) #	0 (0%)	0 (0%)	0 (0%)	1 (7.1%) #
BUT grade OD	13.9 ± 6.1	14.7 ± 5.9	13.9 ± 6.6	16.0 ± 5.0	14.0 ± 6.4	10.4 ± 5.3
BUT grade OS	15.4 ± 5.3	13.6 ± 5.9 #	15.6 ± 6.0	14.4 ± 4.8	14.6 ± 4.6	8.5 ± 5.6 #
OSDI score	4.73 ± 7.79	4.73 ± 7.47	3.58 ± 5.69	2.79 ± 6.09	4.14 ± 5.64	5.13 ± 10.16
** Available measurements OD/OS **	130/129	117/116	61/61	13/13	22/23	6/6
Schirmer test OD (mm/5 min)	17.6 ± 12.1	20.8 ± 11.8 #	21.2 ± 12	26.9 ± 9.5 #	19.3 ± 13	24.5 ± 12.1
Schirmer test OS (mm/5 min)	16.2 ± 11.3	20.1 ± 11.7 #	20.7 ± 11.7 #	28.5 ± 9.6 #	21.1 ± 12.5	23.5 ± 12.1
Schirmer test ≤ 10 mm/5 min OD + OS	102 (39.2%)	64 (27.2%) #	31 (24.8%) #	1 (3.8%) #	12 (26.7%) #	2 (18.2%) #
Schirmer test ≤ 5 mm/5 min OD + OS	52 (20%)	29 (12.3%) #	15 (12%) #	0 (0%) **	7 (15.6%)	2 (18.2%)
** Bulbar redness **						
Valid measurements participants OD/OS	81/76	71/69	42/39	9/6	16/13	6/3
Global bulbar redness OD	0.82 ± 0.32	0.87 ± 0.4	0.87 ± 0.61	0.92 ± 0.32	1.15 ± 0.53 #	1.08 ± 0.43
Global bulbar redness OS	0.72 ± 0.31	0.96 ± 0.56 #	0.81 ± 0.59	0.97 ± 0.56	1.08 ± 0.89	0.9 ± 0.2
Bulbar temporal OD	0.7 ± 0.33	0.78 ± 0.35	0.75 ± 0.43	0.86 ± 0.32	0.92 ± 0.38 #	1.0 ± 0.4
Bulbar temporal OS	0.66 ± 0.28	0.79 ± 0.38 #	0.69 ± 0.34	0.78 ± 0.38	0.78 ± 0.34	1.03 ± 0.15 #
Bulbar nasal OD	1.01 ± 0.4	0.96 ± 0.4	0.88 ± 0.48	1.12 ± 0.39	1.52 ± 0.78 #	1.17 ± 0.52
Bulbar nasal OS	0.91 ± 0.45	1.05 ± 0.52	0.92 ± 0.49	1.42 ± 0.7	1.27 ± 0.82	1.13 ± 0.32
Limbal temporal OD	0.37 ± 0.28	0.43 ± 0.23	0.39 ± 0.28	0.62 ± 0.45	0.53 ± 0.3 #	0.52 ± 0.34
Limbal temporal OS	0.39 ± 0.22	0.5 ± 0.31 #	0.44 ± 0.29	0.5 ± 0.35	0.47 ± 0.29	0.53 ± 0.23
Limbal nasal OD	0.53 ± 0.3	0.5 ± 0.27	0.5 ± 0.35	0.61 ± 0.28	0.79 ± 0.48	0.67 ± 0.33
Limbal nasal OS	0.47 ± 0.31	0.58 ± 0.38	0.49 ± 0.39	0.9 ± 0.81	0.68 ± 0.53	0.87 ± 0.55

GA—gestational age; ROP—retinopathy of prematurity; mm—millimeter; OD—right eye; OS—left eye; BUT—Break-up time. Linear regression analysis was applied for normally distributed parameters to compare the different groups with the full-term control group (reference). When parameters were not normally distributed, Mann–Whitney U-test was conducted to compare the different preterm groups with the full-term control group (reference group). # statistical difference (*p* < 0.05) compared to the control group. ** statistical difference (*p* < 0.001) compared to the control group.

**Table 3:** Lid parameters of the study groups.

	Group 1	Group 2	Group 3	Group 4	Group 5	Group 6
Gestational Age [Weeks]	GA ≥ 37	GA 33–36	GA 29–32	GA ≤ 28	GA ≤ 32	GA ≤ 32
		no ROP	no ROP	no ROP	ROP	Treated ROP
Available lid measurements Participants/eyes (n)	134/268	125/245	64/125	14/28	29/50	8/15
Palpebral fissure (mm) OD	9.52 ± 1.11	9.41 ± 1.43	9.39 ± 1.19	9.25 ± 1.03	9.5 ± 1.17	8.85 ± 1.97
Palpebral fissure (mm) OS	9.17 ± 1.19	8.98 ± 1.56	9.11 ± 1.27	8.61 ± 1.41	9.36 ± 2.35	8.39 ± 2.34
Nasal palpebral angle (degree) OD	53.86 ± 6.83	52.46 ± 7.62	52.92 ± 8.78	51.43 ± 8.48	51.8 ± 6.41	47.88 ± 10.52
Nasal palpebral angle (degree) OS	53.47 ± 7.42	51.72 ± 7.6	52.49 ± 8.6	50.79 ± 6.93	47.68 ± 5.39	46.86 ± 13.23
Bulbar area OD	11.3 ± 4.3	10.9 ± 5.0	11.1 ± 4.9	10.4 ± 3.1	9.6 ± 2.5 #	8.8 ± 3.6
Bulbar area OS	11.2 ± 4.6	9.8 ± 5.2	10.3 ± 5.2	9.7 ± 4.1	9.4 ± 4.4	8.1 ± 2.3 #

GA—gestational age; ROP—retinopathy of prematurity; mm—millimeter; OD—right eye; OS—left eye. Linear regression analysis was applied to compare the different groups with the full-term control group (reference). Categorical data were compared using the chi-square test with the full-term group as reference. # statistical difference (*p* < 0.05) compared to the control group.

**Table 4:** Linear associations of anterior segment parameters with different perinatal parameters for the sample of the Gutenberg Prematurity Eye Study.

First Break-Up Time < 20 s (yes)	Univariate		Model 1		Model 2	
	OR [95% CI]	*p*	B [95% CI]	*p*	B [95% CI]	*p*
Gestational age (weeks)	0.981 (0.947; 1.017)	0.29	-	-	-	-
Birth weight (kg)	0.878 (0.750; 1.028)	0.88	*	*	*	*
Birth weight percentile	0.994 (0.988; 1.000)	0.057	-	-	-	-
ROP (yes)	1.813 (1.083; 3.036)	0.024	-	-	1.425 (0.783; 2.591)	0.25
ROP treatment (yes)	4.711 (1.463; 15.17)	0.009	-	-	4.421 (1.200; 16.28)	0.025
Placental insufficiency (yes)	1.204 (0.617; 2.349)	0.59	-	-	-	-
Preeclampsia (yes)	1.389 (0.896; 2.153)	0.14	-	-	-	-
Breastfeeding (yes)	0.962 (0.711; 1.301)	0.80	-	-	-	-
Smoking pregnancy (yes)	1.144 (0.623; 2.103)	0.66	-	-	-	-
** Bulbar redness **	**B [95% CI]**	***p***	**B [95% CI]**	***p***	**B [95% CI]**	***p***
Gestational age (weeks)	−0.018 (−0.03; −0.006)	0.004	−0.019 (−0.030; −0.008)	<0.001	−0.015 (−0.027; −0.003)	0.011
Birth weight (kg)	−0.076 (−0.127; −0.026)	0.003	*	*	*	*
Birth weight percentile	−0.002 (−0.003; 0)	0.098	-	-	-	-
ROP (yes)	0.202 (−0.001; 0.405)	0.05	-	-	0.108 (−0.102; 0.319)	0.31
ROP treatment (yes)	0.147 (−0.097; 0.390)	0.24	-	-	-	-
Placental insufficiency (yes)	−0.067 (−0.255; 0.121)	0.49	-	-	-	-
Preeclampsia (yes)	0.055 (−0.098; 0.207)	0.48	-	-	-	-
Breastfeeding (yes)	−0.087 (−0.193; 0.018)	0.10	-	-	-	-
Smoking pregnancy (yes)	−0.135 (−0.328; 0.058)	0.17	-	-	-	-
** Wetting Schirmer test (mm) **	**B [95% CI]**	***p***	**B [95% CI]**	***p***	**B [95% CI]**	***p***
Gestational age (weeks)	−1.067 (−1.638; −0.496)	<0.001	−0.692 (−1.158; −0.226)	0.003		
Birth weight (kg)	−4.918 (−7.330; −2.506)	0.001	*	*		
Birth weight percentile	0.048 (−0.068; 0.163)	0.41	-	-		
ROP (yes)	3.0 (−3.137; 9.137)	0.34	-	-		
ROP treatment (yes)	3.0 (−13.203; 19.203)	0.22	-	-		
Placental insufficiency (yes)	12.0 (1.259; 22.74)	0.029	8.596 (4.643; 12.55)	<0.001		
Preeclampsia (yes)	0.0 (−10.73; 10.73)	1.0	-	-		
Breastfeeding (yes)	3.0 (−2.514; 8.515)	0.29	-	-		
Smoking pregnancy (yes)	8.0 (−1.875; 17.87)	0.11	-	-		
** Nasal palpebral angle (degree) **	**B [95% CI]**	***p***	**B [95% CI]**	***p***	**B [95% CI]**	***p***
Gestational age (weeks)	0.266 (0.098; 0.435)	0.002	0.220 (0.050; 0.390)	0.011	0.142 (0.039; 0.323)	0.125
Birth weight (kg)	1.159 (0.418; 1.9)	0.002	*	*	*	*
Birth weight percentile	0.019 (−0.009; 0.048)	0.19	-	-	-	-
ROP (yes)	−3.643 (−5.998; −1.288)	0.002	-	-	−2.384 (−4.986; 0.219)	0.073
ROP treatment (yes)	−5.175 (−11.934; 1.584)	0.13	-	-	-	-
Placental insufficiency (yes)	−0.555 (−4.721; 3.611)	0.79	-	-	-	-
Preeclampsia (yes)	2.31 (0.107; 4.513)	0.040	2.360 (0.177; 4.544)	0.034	2.492 (0.320; 4.664)	0.025
Breastfeeding (yes)	−0.668 (−2.098; 0.762)	0.36	-	-	-	-
Smoking pregnancy (yes)	0.622 (−2.422; 3.667)	0.69	-	-	-	-

B—Beta; CI—confidence interval; mm—millimeter. Linear regression analysis using generalized estimating equations to control for correlations between right and left eyes for normally distributed parameters. Binary logistic regression analyses were used for the parameter BUT < 20 s and quantile regression analyses for wetting of the Schirmer test (mm). Model 1 included sex (female), age (years), and univariate associated factors ≤ 0.05 but not ROP occurrence and treatment. * Birth weight (kg) was not included in this model due to the high correlation with gestational age. Model 2 included sex (female), age (years), gestational age (weeks), and univariate associated factors ≤ 0.05 and ROP occurrence and ROP treatment (if associated in univariate analyses).

## Data Availability

A.F. had full access to all study data and takes responsibility for the integrity of the data and the accuracy of the data analysis. Statistical analyses were performed by A.F. The analysis presents clinical data of a cohort. This project constitutes a major scientific effort with high methodological standards and detailed guidelines for analysis and publication to ensure scientific analyses are on the highest level; therefore, data are not made available for the scientific community outside the established and controlled workflows and algorithms. To meet the general idea of verification and reproducibility of scientific findings, we offer access to data at the local database upon request at any time. Interested researchers may make their requests to the coordinating PI of the GPES (Achim Fieß; achim.fiess@unimedizin-mainz.de). More detailed contact information is available at the homepages of the UM (www.unimedizin-mainz.de).

## Supplementary Materials

The following supporting information can be downloaded at: https://www.mdpi.com/article/10.3390/jcm11102702/s1, Figure S1: Gutenberg Prematurity Eye Study design.

## Abbreviations

GA	Gestational age
GCP	Good Clinical Practice
GEE	General estimating equations
GEP	Good Epidemiological Practice
GPES	The Gutenberg Prematurity Eye Study
OSDI	Ocular Surface Disease Index
ROP	Retinopathy of prematurity
UMCM	University Medical Center of the Johannes Gutenberg University Mainz in Germany
