# Supplementary figures and images for: Dry Eye Parameters and Lid Geometry in Adults Born Extremely, Very, and Moderately Preterm with and without ROP: Results from the Gutenberg Prematurity Eye Study
Source: J Clin Med. 2022 May 11;11(10):2702. doi: 10.3390/jcm11102702 (PMC9147172; doi:10.3390/jcm11102702)

## Supplementary Figure S1

### Gutenberg Prematurity Eye Study design.

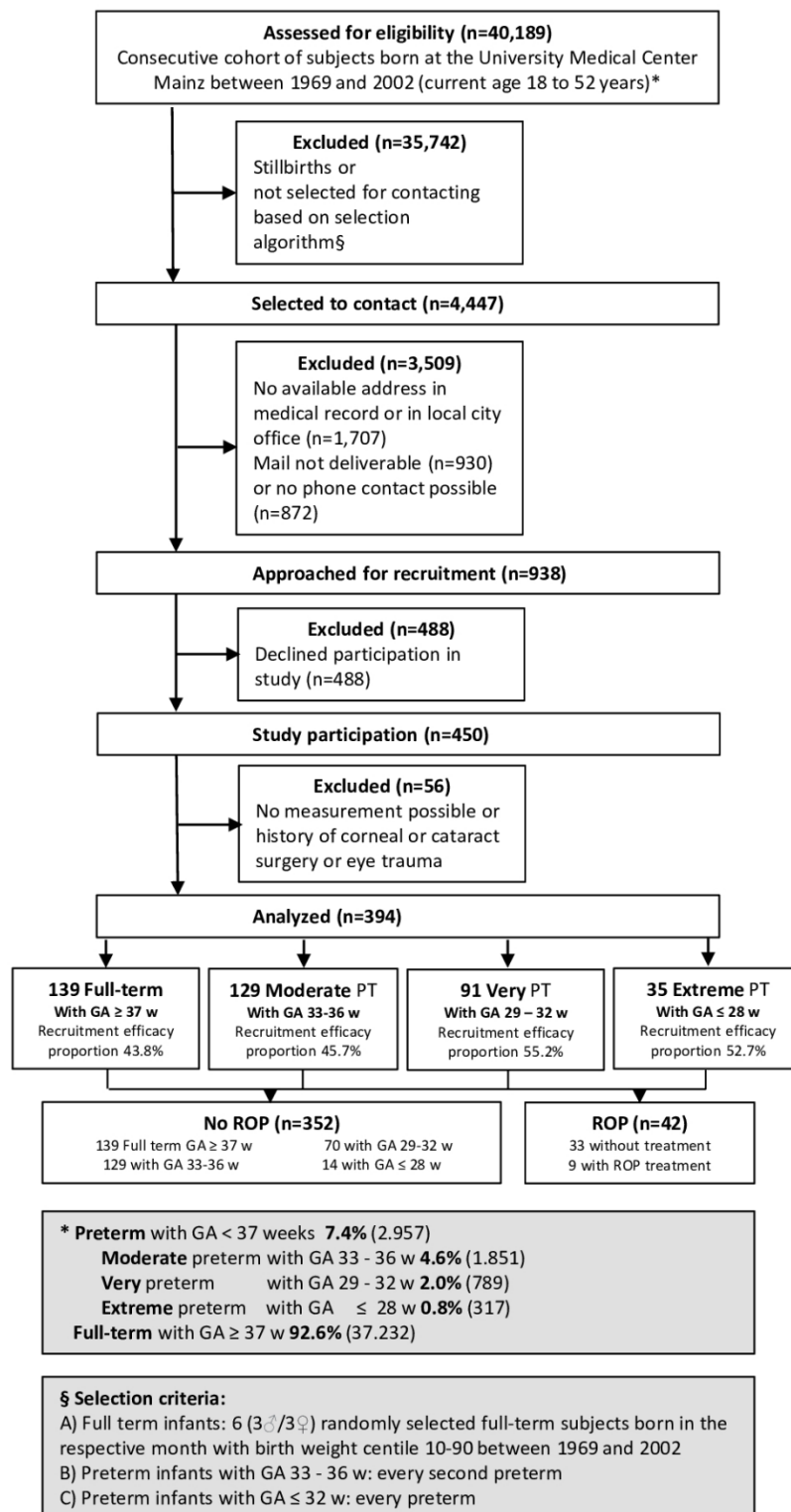

Supplement: Supplementary file 1 [file jcm-11-02702-s001.zip › jcm-1660870-supplementary.pdf]
